# Soluble endoglin as a biomarker of successful rheopheresis treatment in patients with age-related macular degeneration

**DOI:** 10.1038/s41598-024-80375-5

**Published:** 2024-11-21

**Authors:** Vladimir Blaha, Jana Urbankova Rathouska, Hana Langrova, Milan Blaha, Jan Studnicka, Ctirad Andrys, Veronika Loefflerova, Miriam Lanska, Eva Vejrazkova, Petr Nachtigal, Alexandr Stepanov

**Affiliations:** 1 Faculty of Medicine in Hradec Kralove, 3rd Department of Internal Medicine - Metabolism and Gerontology, University Hospital Hradec Kralove and Charles University, Hradec Kralove, Czech Republic; 2 Department of Biological and Medical Sciences, Faculty of Pharmacy in Hradec Kralove, Charles University, Heyrovskeho 1203, 500 05 Hradec Kralove, Czech Republic; 3 Faculty of Medicine in Hradec Kralove, Department of Ophthalmology, University Hospital Hradec Kralove and Charles University, Hradec Kralove, Czech Republic; 4 Faculty of Medicine in Hradec Kralove, 4th Department of Internal Medicine - Hematology, University Hospital Hradec Kralove and Charles University, Hradec Kralove, Czech Republic; 5 Department of Immunology and Allergology, Faculty of Medicine in Hradec Kralove, University Hospital Hradec Kralove and Charles University, Hradec Kralove, Czech Republic; 6 Department of Ophthalmology, Regional Hospital Liberec, Liberec, Czech Republic

**Keywords:** Age-related macular degeneration, Rheopheresis, Soluble endoglin, Alpha-2-macroglobulin, Biomarkers, Health care, Medical research

## Abstract

Age-related macular degeneration (AMD) is a progressive chronic disease causing visual impairment or central vision loss in the elderly. We hypothesized that successful rheopheresis would be associated with positive changes in soluble endoglin (sENG), PSCK9, alpha-2-macroglobulin (A2M), and hs-CRP levels. 31 elderly patients with the dry form of AMD, treated with rheopheresis with a follow-up period of at least 5 years and an average age of 68 ± 4 years, were evaluated. Each treated patient received a series of 8 procedures in 10 weeks and, after the 2-year period, another 2 procedures within 1 week. Then, the patients were followed up every 6 months and divided into the successfully treated and therapeutic failure group according to best-corrected visual acuity (BCVA), size of the drusen area, and the drusenoid pigment epithelium detachment (DPED). Based on the ophthalmological assessment, rheopheresis treatment was successful in 73% of AMD patients. The therapy was associated with a significant decrease in total cholesterol, LDL-C, HDL-C, apoprotein B, lipoprotein (a) levels, and rheologically important parameters, irrespective of the therapy’s success or failure. The success of rheopheresis therapy was exclusively related to a significant decrease in sENG and A2M levels. Over the long term, rheopheresis prevented the decline of BCVA, reduced the DPED and area of macular drusen, and improved the preservation of an intact photoreceptor ellipsoid zone in most patients. Moreover, we showed for the first time that sENG and A2M could be potentially sensitive biomarkers of successful rheopheresis procedure, irrespective of lipid parameters changes.

## Introduction

Age-related macular degeneration (AMD) is a progressive chronic disease, and despite the best care, it remains a leading cause of visual impairment in the elderly. The progression of AMD involves the transition from an early or intermediate stage, when extracellular deposits called drusen accumulate on the inner surface of Bruch’s membrane, to an advanced stage featuring photoreceptor and retina pigment epithelium (RPE) atrophy and/or choroidal neovascularization (CNV), leading to central vision loss^1^. A hypothesis implicating hyperlipidemia/hypercholesterolemia as a risk factor in AMD has arisen from the observation that AMD and cardiovascular disease share several risk factors and pathophysiological pathways^2^. The retina has been shown to uptake circulating low-density lipoprotein (LDL)^3^, providing blood-borne lipids to all the cellular layers of the retina. The retina can also synthesize cholesterol to maintain its dynamic steady-state lipid composition^4^. To perform these tasks, the retina expresses the same molecules used in blood-borne lipoprotein uptake and in the intraretinal lipoprotein-based lipid transport process, including the proprotein convertase subtilisin/kexin type-9 (PCSK9)^5^.

Moreover, several studies showed the relationship between AMD and inflammation (increased hs-CRP) and endothelial dysfunction development^6^. Also, the complement system (alpha-2-macroglobulin (A2M)) was found to be participating in the development of dysfunctional RPE or choroidal blood vessels^7^.

Endoglin (ENG, CD105), a 180 kDa transmembrane glycoprotein, is considered a co-receptor for ligands of the transforming growth factor β (TGFβ) superfamily. There are two isoforms currently described. The membrane (tissue) ENG is expressed by various cells and soluble endoglin (sENG) circulating in plasma or cell culture medium^8^. sENG is the cleavage product of the extracellular domain of ENG formed by the activity of matrix metalloproteinase 14 (MMP-14)^9^ that is released into the circulation. sENG was demonstrated to be a possible biomarker of various cardiovascular and metabolic disorders, such as familial hypercholesterolemia^10,11^, arterial hypertension^12^, preeclampsia^13^, and endothelial dysfunction/atherosclerosis^14^. In addition, high levels of sENG were demonstrated to aggravate endothelial dysfunction together with hypercholesterolemia^15^. However, sENG role in AMD has not been studied so far.

Rheopheresis (a modification of lipoprotein apheresis) is an extracorporeal membrane filtration method for the elimination of high molecular weight proteins (i.e., fibrinogen, immunoglobulin M, thrombomodulin), which also significantly decreases low-density lipoprotein cholesterol (LDL)^16^. This method can normalize rheologically important parameters (the viscosity of blood and plasma, as well as erythrocyte aggregability), improve erythrocyte flexibility, and could lead to substantial improvement of visual functions in people suffering from AMD^17^. Consequently, this could improve blood flow in the choroid, which is reduced in the dry form of AMD^18^. Indeed, many studies have demonstrated the effectiveness of rheopheresis in most AMD patients^19–23^.

Interestingly, it has previously been demonstrated that lipoprotein apheresis can reduce endothelial dysfunction and inflammation biomarkers, including sENG and hsCRP, not only after each procedure but also in long-term monitoring in familial hypercholesterolemia patients^24^. In addition, it was demonstrated that rheopheresis decreases A2M in AMD patients^25^ and PCSK9 levels in familial hypercholesterolemia patients after lipoprotein apheresis^26^.

Currently, there are no studies that evaluated the rheopheresis effect on sENG, PCSK9, hsCRP, and A2M levels in relation to the success of this therapy in AMD patients. Thus, we aimed to confirm our hypothesis that the effect of rheopheresis (from the ophthalmological point of view) is related to the reduction of sENG, PCSK9, hsCRP, and A2M levels only in successfully treated AMD patients.

## Materials and methods

The project was an open-labeled study involving patients with AMD. We followed 31 patients with AMD using rheopheresis between March 2012 and April 2021. The original study has been registered under Clinical Trial Registration number NCT01943396 (17/09/2013). The study’s sample size and power calculations were based on the primary efficacy endpoint. Results from previous studies were used to estimate the mean change expected. It was estimated that the enrolment of 31 patients would provide the study with a statistical power of 1 − β = 80% and a 2-sided alpha = 0.05 to detect a difference between the two study groups.

Out of 31 patients in this study, 26 patients who completed rheopheresis treatment using the predetermined scheme, with at least a 5-year follow-up (mean 6.33 years, median 6.25 years, and range of follow-up duration 5.00–8.92 years), were included for evaluation. The patients were further divided into two groups based on the results of the ophthalmological examination. The first group consisted of patients who were clinically successfully treated, and the second group included patients with therapeutic failure. Patients whose disease became stable or improved (best corrected visual acuity (BCVA) and morphological findings on the posterior eye segment) were considered as being successfully treated. (Note: the group of successfully treated patients is numerically larger since it includes patients who were found to have improved and those with stable disease. Generally, long-term stabilization is considered a successful therapy in the case of a dry form of AMD). Among 26 patients who completed rheopheresis treatment using the predetermined scheme, 19 patients were evaluated as successfully treated and seven patients as treatment failed.

### Inclusion/exclusion criteria

Based on our long-term experiences, we stipulated the indication criteria and contraindications of treatment of AMD by rheopheresis.

Inclusion criteria for treatment by rheopheresis were the presence of a dry form of AMD with soft drusen in stage 1-3 according to the EURYEYE study^27^ with BCVA within the range of 85–45 (monocular 40) letters of ETDRS optotypes. Another inclusion criterion was body weight over 50 kg and other feasible indications for apheresis therapy (peripheral veins allowing vascular access to establish the extracorporeal circuit).

Exclusion criteria were the presence of a wet form of AMD, geographic atrophies of the RPE in the macula or other irreversible changes, pathologies of the retina and choroid other than AMD (dystrophy, inflammatory diseases), new macular hemorrhage on the ocular fundus, pathology of the optic nerve including glaucoma and opacity of the optic media limiting examination of the posterior eye segment. Other exclusion criteria were uncontrolled diabetes mellitus, uncontrolled arterial hypertension, insufficient antecubital venous access, haemato-oncological malignancies, patients who were unwilling to adhere to the examination visit schedule or who were in poor general condition (serious diseases—infections, cardiovascular or cerebral insufficiency, severe coronary artery disease).

### Ophthalmological examination and clinical evaluation of the patients (visual acuity, morphological ocular findings)

The most important criterion, especially from the patient’s point of view, is visual acuity: this was evaluated as stabilization if the BCVA was within ± 10 letters of the ETDRS optotypes from the baseline on a specified date (i.e., change of a maximum of two lines of the ETDRS optotypes). Improvement of BCVA by ≥ 11 letters of the ETDRS optotypes was evaluated as an improvement; deterioration by ≥ 11 letters was considered worsening. Stabilization of the AMD was considered a change in the original area of pathological changes (area of drusenoid pigment epithelium detachment (DPED)/soft drusen in mm^2^) by ± 20%. A decrease of > 20% in the pathological changes was considered an improvement; an analogical increase of > 20% from the baseline was considered a worsening. An experienced ophthalmologist considered the effect of the therapy. Our approach to the data evaluation^21,23,28^ was published in a series of original manuscripts and later also accepted by the American Society for Apheresis^29^ to create Guidelines on the Use of Therapeutic Apheresis in Clinical Practice^30^.

The patients were followed up every 6 months, at least 5 years. During each visit, the patient underwent the following ophthalmological examinations: BCVA on ETDRS optotype tables, monitoring of the development of soft drusen/DPED [mm^2^] using the VISUPAC program on a digital fundus camera (FF 450 + IR, Zeiss) after manual marking of the area, optical coherence tomography (OCT; Cirrus, Zeiss from 2009; 2005–2009: Stratus, Zeiss) for verification of drusen deposits, determination of retinal thickness and anatomical changes, especially in the region of the ellipsoid photoreceptors. In addition, it was carried out fluorescence angiography (FAG) on a digital fundus camera (FF 450 + IR, Zeiss) from 2019 angio-OCT (Spectralis, Heidelberg) in case of suspicion of deterioration into the wet form of AMD.

### Rheopheresis

Rheopheresis therapy was used according to Borberg et al. with our own modification^17,31^. To obtain plasma, we used continuous separators (Cobe Spectra or Spectra Optia, Terumo BCT, Lakewood, Co, USA) and Evaflux 4A filters (Kawasumi, Tokyo, Japan) to wash the obtained plasma were used. The flow through the filter was controlled using the CF100 automatic machine (Informed, Geneva, Switzerland). Anticoagulation was performed using a combination of heparin and ACD-A (Baxter, Munich, Germany). 1–1.5 l of blood was washed. The procedures were performed from the peripheral vein in the elbow pit or in the forearm. We presented some more detailed data separately^21,23,28^. The treatment scheme was performed for patients with AMD according to the MIRA-1 study^32^; 8 procedures in 10 weeks, i.e., two aphereses weekly at intervals of 2–4 days, followed by a break of 14 days, followed by another series of aphereses. The patients were then followed up every 6 months. According to our experience, the effects of a successful treatment usually last from 2 to 2.5 years. It is recommended (based on the literature data and our experience^16,23^) that 1-2 additional procedures be performed to boost the effect (“booster therapy”) after these 2 years. We decided on two procedures within 1 week for our group of patients. Other important points are the side effects/adverse effects of rheopheresis. These are carefully monitored and were published by our team earlier^33^. Our experience with long-term therapy shows good tolerance and a small number of complications (5, 6% of clinically irrelevant side-effects), which were subsequently resolved by standard symptomatic treatment.

### Plasma samples and blood analysis

Blood samples were collected immediately before and after rheopheresis in EDTA-containing tubes and centrifuged within 30 min at 1500G for 15 min at room temperature. Plasma samples were aliquoted and stored at − 80 °C before the proteomic analysis. The analysis was performed periodically with a consistent methodology. Sampling was done at the baseline before therapy, in the middle of the protocol (after 4th rheopheresis), and at the end of the protocol (after 8th rheopheresis).

TC, LDL-C, HDL-C, and apoprotein B were determined using a commercial kit with a Modular Roche analyzer. Lp(a) was measured using the Lipoprotein(a) RxDx assay (Roche Diagnostics GmbH). Hematological parameters were assessed using routine laboratory techniques (fibrinogen, blood and plasma viscosity).

### Analysis of PCSK9

Serum concentrations of PCSK9 were analyzed using Quantikine Human Proprotein Convertase 9/PCSK9 produced by company R&D Systems (USA). Instructions from the producer were always respected. Serum samples were diluted 20×, and the measurement range was 0.3–40 ng/ml.

### Evaluation of biomarkers of inflammation and endothelial dysfunction

The levels of high-sensitivity C-reactive protein (hsCRP) were assessed by immunonephelometry with analyzer IMMAGE 800 (Beckman, USA), and results were expressed in milligrams per liter (mg/L) of serum with a detection limit of 1.0 mg/L.

The levels of alpha-2-macroglobulin (A2M) were assessed by immunonephelometry on IMMAGE 800 (Beckman, USA), and results were expressed in milligrams (mg) per deciliter (dL) with a detection limit of 20 mg/dL.

The concentrations of soluble endoglin (CD105, sENG) were assessed in serum samples by sandwich enzyme-linked immunosorbent assay technique (ELISA) using the Quantikine Human Endoglin/CD105 ELISA kit (R&D Systems, MN, USA) according to the manufacturer’s instructions. Samples were undiluted. The sensitivity of the kit was 0.007 ng/mL. The absorbance values were measured at 450 nm with a Multiskan RC ELISA reader (Thermo Fisher Scientific, MA, USA).

### Statistical analyses

Data are presented as median with interquartile range, excluding absolute and relative patient frequencies. Wilcoxon matched-paired signed rank tests were used for intergroup comparisons and t-test for evaluation of basic clinical data of patients. A value of *p* < 0.05 was the minimum requirement for a statistically significant difference. GraphPad Prism software version 9.2 (GraphPad Software Inc., San Diego, CA, USA) was used for the statistical analyses.

## Results

### Clinical characteristics of the patients

The group of successfully treated (average age was 68.68 ± 4.62 years) consisted of 12 females and seven males. The successfully treated patients (see Table 1) had a diagnosis of coronary artery disease, n = 2 (10%); cerebrovascular disease, n = 1 (5%); peripheral artery disease, n = 1 (5%); diabetes mellitus, n = 1 (5%); hypertension, n = 9 (47%); hyperlipoproteinemia, n = 9 (47%). Most of the patients diagnosed with hyperlipidemia in successfully treated AMD patients (n = 7) were on long-term therapy by statin, which has been started before the treatment of AMD using rheopheresis (daily use of simvastatin 20 mg, atorvastatin 10 mg, atorvastatin 20 mg, rosuvastatin 10 mg and/or rosuvastatin 15 mg). There were two patients diagnosed with hyperlipidemia in this group who did not tolerate any available hypolipidemic therapy.

**Table 1:** Clinical data in successfully treated AMD patients and in patients with therapeutic failure.

	Successfully treated	Therapeutic failure	P
Age (mean ± SD)	68.68 ± 4.62	68.29 ± 4.39	NS
Sex (F/M)	12/7	5/2	*p* < 0.05
CAD patients (%)	2 (10)	0 (0)	*p* < 0.05
CVD patients (%)	1 (5)	0 (0)	*p* < 0.05
PAD patients (%)	1 (5)	0 (0)	*p* < 0.05
DM patients (%)	1 (5)	0 (0)	*p* < 0.05
H patients (%)	9 (47)	4 (57)	NS
HLP patients (%)	9 (47)	3 (43)	NS
Statin patients (%)	7 (37)	3 (43)	NS

Results are in successfully treated patients and patients with therapeutic failure.

*F* female, *M* man, *CAD* coronary artery disease, *CVD* cerebrovascular disease, *PAD* peripheral artery disease, *DM* diabetes mellitus, *H* hypertension, *HLP* hyperlipidemia.

The second group included patients with therapeutic failure (Table 1). The average age was 68.29 ± 4.39 years. This group consisted of five females and two males. None of the patients was treated for coronary artery disease, cerebrovascular disease, peripheral artery disease, or diabetes mellitus. Hypertension was present in four patients (57%), and hyperlipoproteinemia in three patients (43%). All patients diagnosed with hyperlipidemia in the group with therapeutic failure (n = 3) were on long-term therapy by statin (daily use of simvastatin 10 mg, atorvastatin 10 mg, atorvastatin 20 mg). This statin therapy has also started before the treatment of AMD using rheopheresis. Cataract surgery was performed in all patients before the study, and no complications in artificial lenses were observed.

### Rheopheresis improves visual acuity and morphological ocular findings in most of the patients

A detailed course of ophthalmological examination was published earlier by our team^21,28,34^. The study eyes were homogeneous for the baseline BCVA area of DPED/drusen, except for significantly higher BCVA and the significantly larger area of macular drusen in the second group (therapeutic failure). A total of 26 eyes were included in the study (17 right eyes and nine left eyes; in three patients in whom we used left eyes, a wet form of AMD was present on their right eyes at baseline, and three patients developed a tight epiretinal membrane during the follow-up on their right eyes). In one patient, we observed progression into a wet form AMD in the study eye during the follow-up period. In the case of the other two patients, geographic RPE atrophy and ellipsoid defects in the study eye were developed.

At baseline, mean BCVA was 76.42 ± 6.13 letters of ETDRS optotypes for the whole cohort of patients, specifically 74.84 ± 5.73 letters for patients in the first group (successfully treated), and 80.71 ± 5.35 letters in the second group (therapeutic failure). The difference of BCVA between groups at baseline was statistically significant (*p* < 0.05). After 5 years, the mean BCVA remained practically stable at a level of 72.62 ± 11.72 in the whole cohort of patients and in the first group (77.0 ± 7.37 letters). In contrast, it decreased significantly to 60.71 ± 13.55 in the second group (*p* < 0.05), so the differences between groups of patients were only nonsignificant (*p* = 0.81). After 5 years of follow-up, BCVA improvement of > 11 ETDRS letters was present in 2/19 patients in the first group, while a BCVA decrease of > 11 letters was noticed in 5/7 patients in the second group. All remaining patients had changes of BCVA within ± 10 ETDRS letters.

The average baseline value of the area of macular drusen/DPED was 11.29 ± 8.34 mm^2^ for the whole cohort, with an average of 11.3 ± 7.67 mm^2^ for patients in the first group and 11.25 ± 10.65 mm^2^ in the second group. The difference in the area of macular drusen between groups at baseline was statistically significant (*p* < 0.05). The area of macular drusen at the end of the five-year follow-up period was 9.5 ± 7.67 mm^2^ for the whole cohort, specifically 8.86 ± 5.86 mm^2^ for patients in the first group, 11.24 ± 11.7 mm^2^ in the second group (*p* < 0.05).

### The rheopheresis treatment scheme reduces total cholesterol, LDL-C, HDL-C, apoprotein B, lipoprotein (a) levels, and rheologically important parameters in AMD patients

**Fig. 1:**
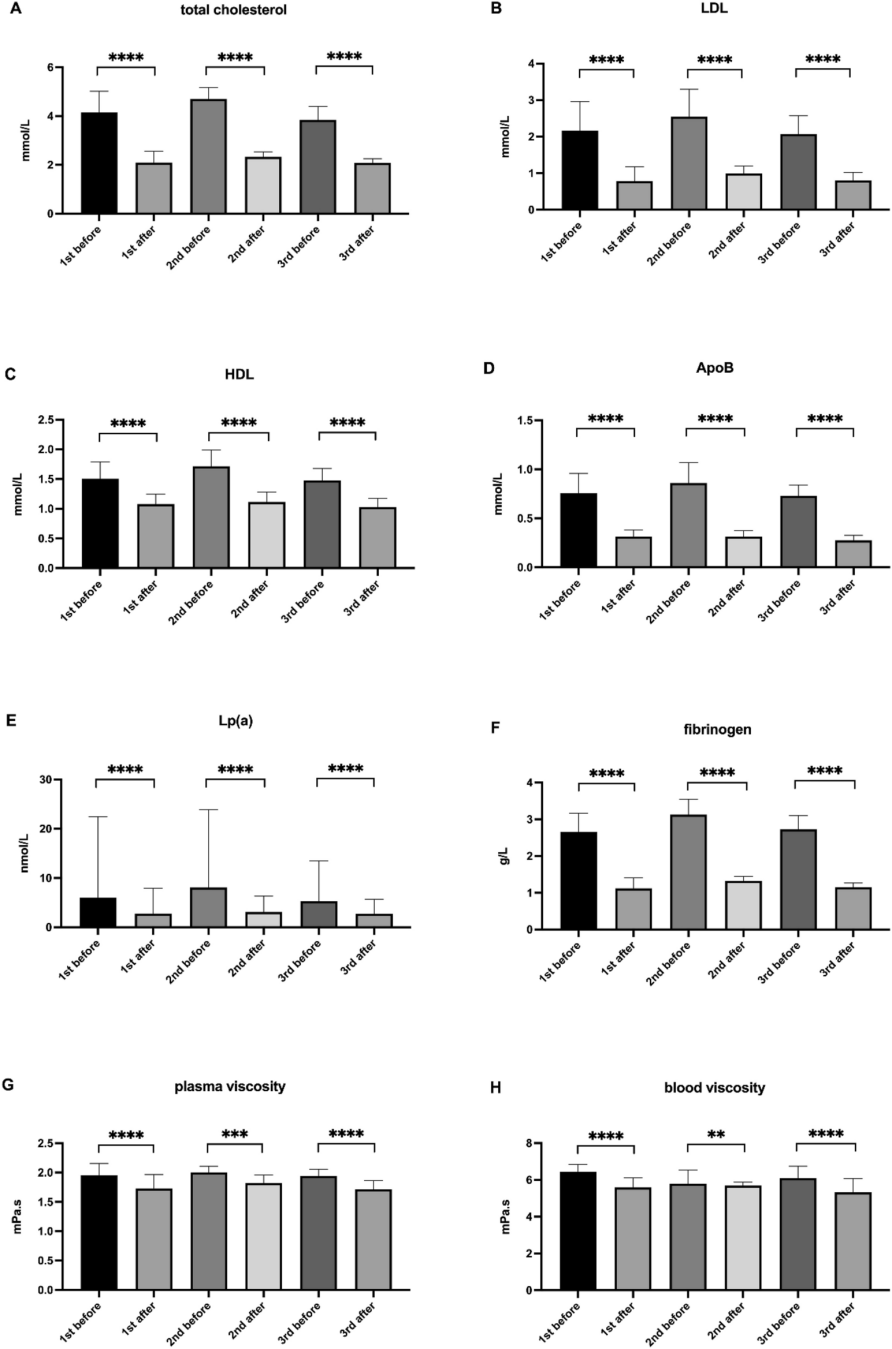
The effect of rheopheresis treatment on lipid and rheologically important parameters. Total cholesterol (**A**), LDL (**B**), HDL (**C**), ApoB (**D**), Lp(a) (**E**), fibrinogen (**F**), plasma viscosity (**G**), blood viscosity (**H**) data are shown as median with interquartile range. Wilcoxon matched-paired signed rank tests were used in the first (1st), the second (2nd), and the third (3rd) rheopheresis treatment before and after therapy*,* ns *p* ≥ 0.05, *p < 0.05, **p < 0.01, ***p < 0.001, ****p < 0.0001.

The analysis of the rheopheresis effect was evaluated in all treated patients. Every single rheopheresis significantly decreased all lipid and rheologically important parameters, as analyzed before and after the first (1st before and 1st after), the second (2nd before and 2nd after), and the third (3rd before and 3rd after) rheopheresis procedure (Fig. 1). The evaluated parameters of lipid profile consisted of total cholesterol (Fig. 1A), LDL cholesterol (Fig. 1B), HDL cholesterol (Fig. 1C), ApoB (Fig. 1D) and Lp(a) (Fig. 1E). The evaluated rheologically important parameters included blood fibrinogen (Fig. 1F), plasma viscosity (Fig. 1G) and blood viscosity (Fig. 1H).

### sENG, PCSK9, and markers of inflammation are reduced in rheopheresis procedures

**Fig. 2:**
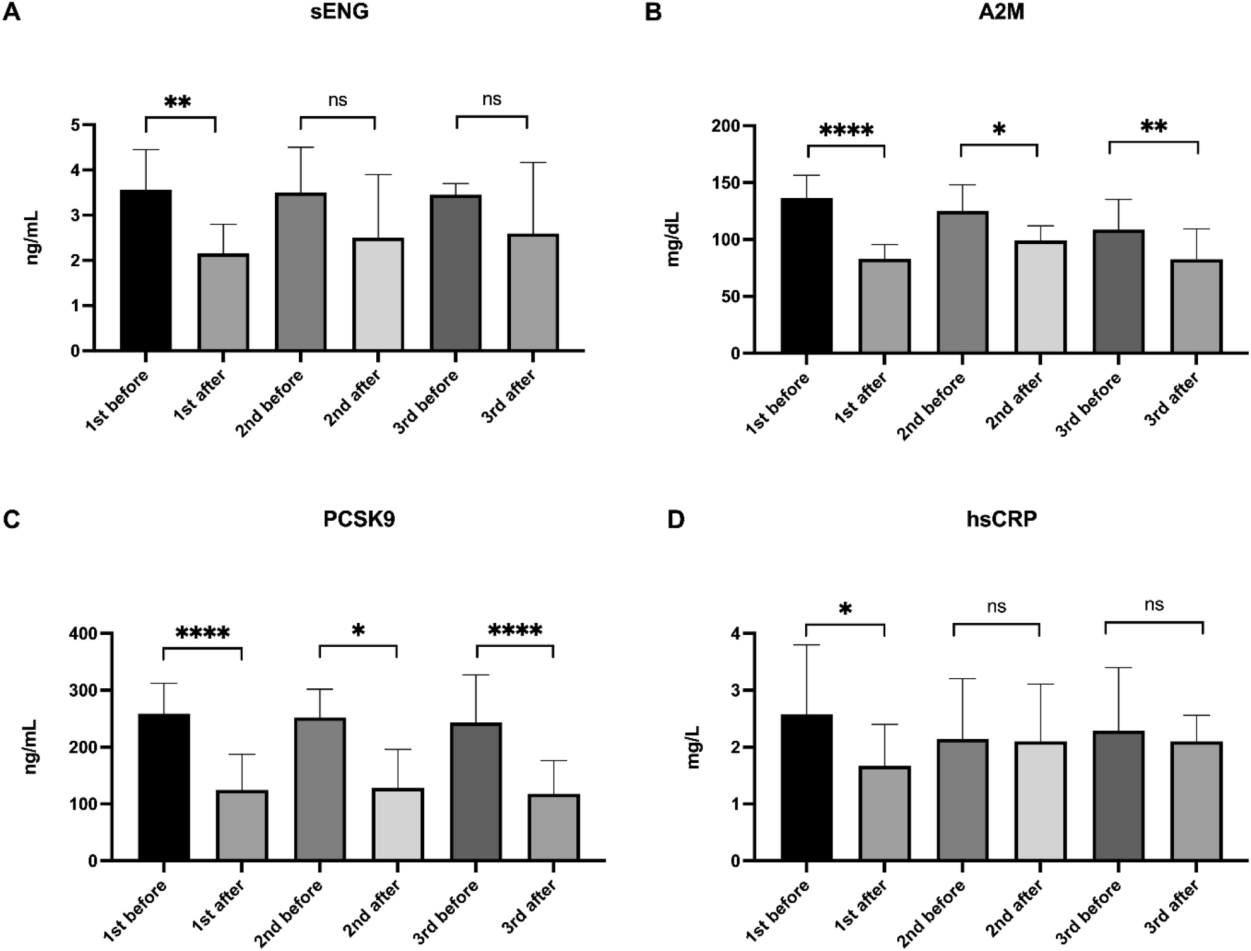
The effect of rheopheresis treatment on sENG (**A**), PCSK9 (**C**), and markers of inflammation A2M (**B**), and hsCRP (**D**). All data are shown as median with interquartile range. Wilcoxon matched-paired signed rank tests were used in the first (1st), the second (2nd), and the third (3rd) rheopheresis treatment before and after therapy, ^ns^ *p* ≥ 0.05, *p < 0.05, **p < 0.01, ****p < 0.0001.

The analysis of the rheopheresis effect on sENG, PCSK9, and markers of inflammation was evaluated in all treated patients (Fig. 2). All three procedures of rheopheresis significantly decreased levels of PCSK9 (Fig. 2C) and A2M (Fig. 2B), as analyzed before and after the first (1st before and 1st after), the second (2nd before and 2nd after) and the third (3rd before and 3rd after) rheopheresis. The rheopheresis effect was also visible in significantly reduced levels of sENG and hsCRP after 1st rheopheresis (Fig. 2A, D, respectively).

### Patients with therapeutic success and therapeutic failure do not significantly differ in lipid and rheologically important parameters

**Fig. 3:**
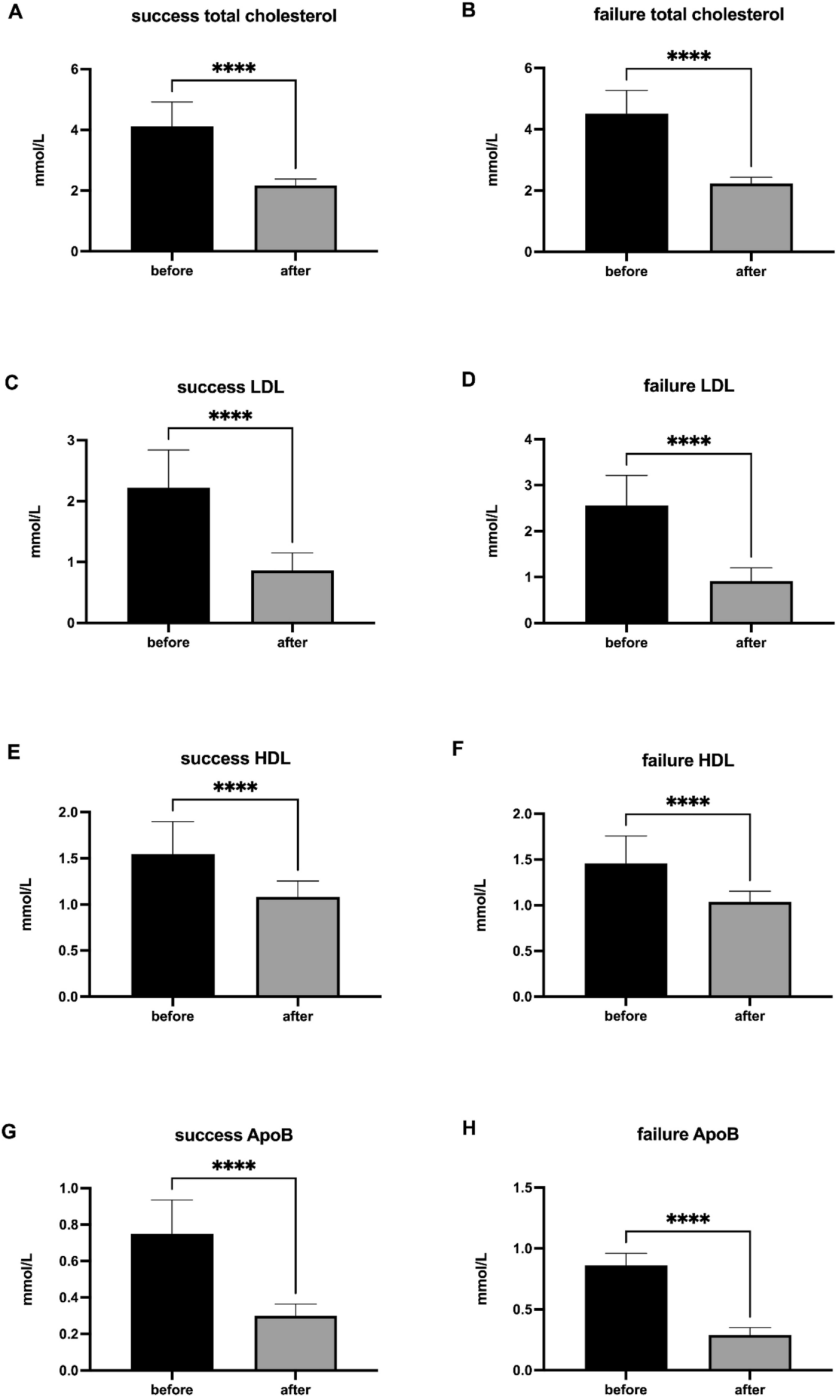

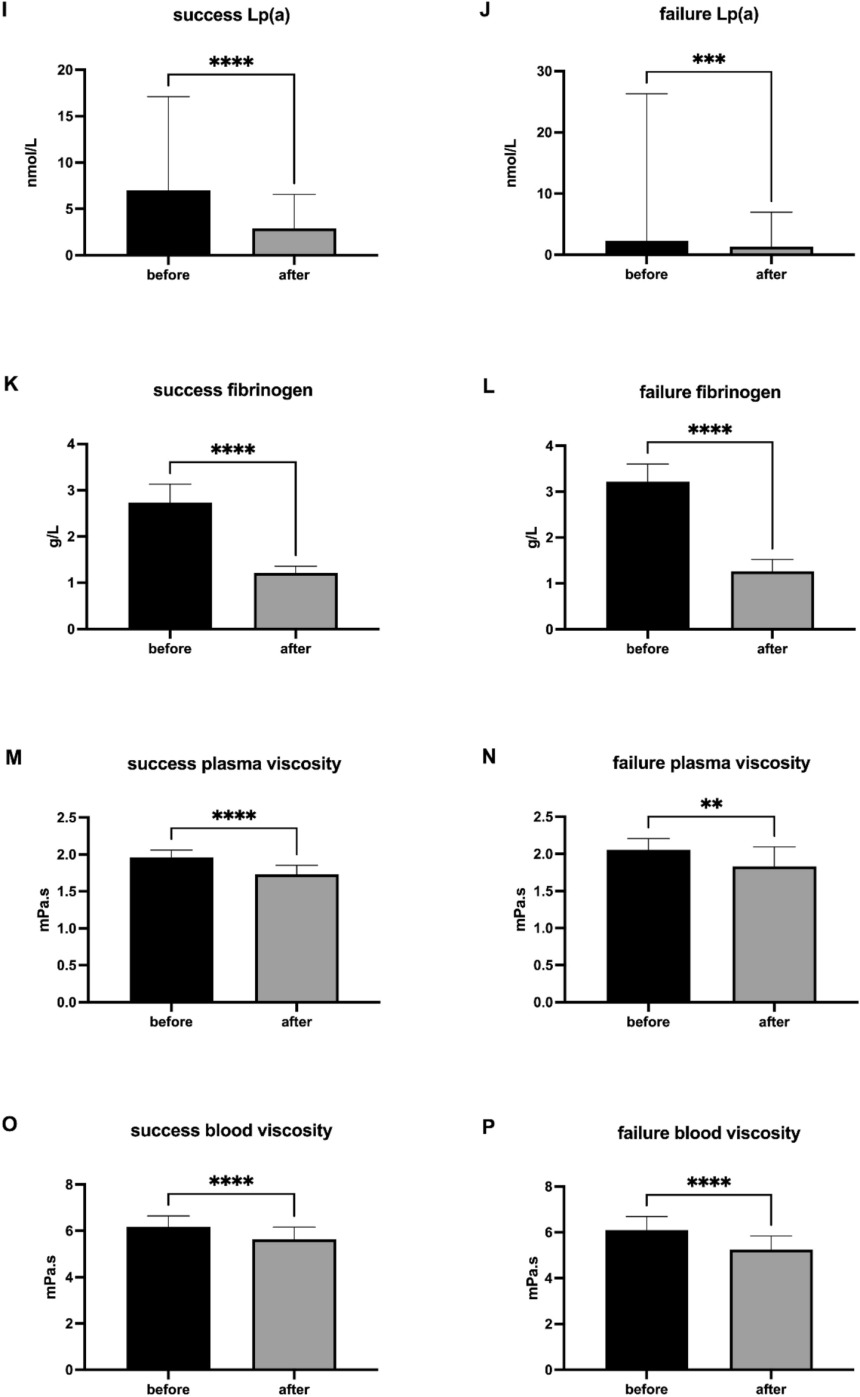
Lipid and rheologically important parameters in patients with therapeutic success and therapeutic failure. Success total cholesterol (**A**), failure total cholesterol (**B**), success LDL (**C**), failure LDL (**D**), success HDL (**E**) failure HDL (**F**), success ApoB (**G**), failure ApoB (**H**), success Lp(a) (**I**), failure Lp(a) (**J**), success fibrinogen (**K**), failure fibrinogen (**L**), success plasma viscosity (**M**), failure plasma viscosity (**N**), success blood viscosity (**O**), failure blood viscosity (**P**) data are shown as median with interquartile range. Wilcoxon matched-paired signed rank tests were used in the first (1st), the second (2nd), and the third (3rd) rheopheresis treatment before and after therapy*,* **p < 0.01, ***p < 0.001, ****p < 0.0001.

As assigned by the ophthalmological assessment (Fig. 3), the changes in lipid and rheologically important parameters before and after rheopheresis procedures were evaluated in the group of successfully treated patients, and the group of therapeutic failure. Rheopheresis procedures had a significant impact on lowering lipid parameters—total cholesterol (Fig. 3A, B), LDL cholesterol (Fig. 3C, D), HDL cholesterol (Fig. 3E, F), ApoB (Fig. 3G, H), Lp(a) (Fig. 3I, J) and lowering of rheological parameters—fibrinogen (Fig. 3K, L), plasma viscosity (Fig. 3M, N) and blood viscosity (Fig. 3O, P), irrespective of the success or the failure of the therapy.

### sENG and A2M are biomarkers of successfully treated patients with AMD

**Fig. 4:**
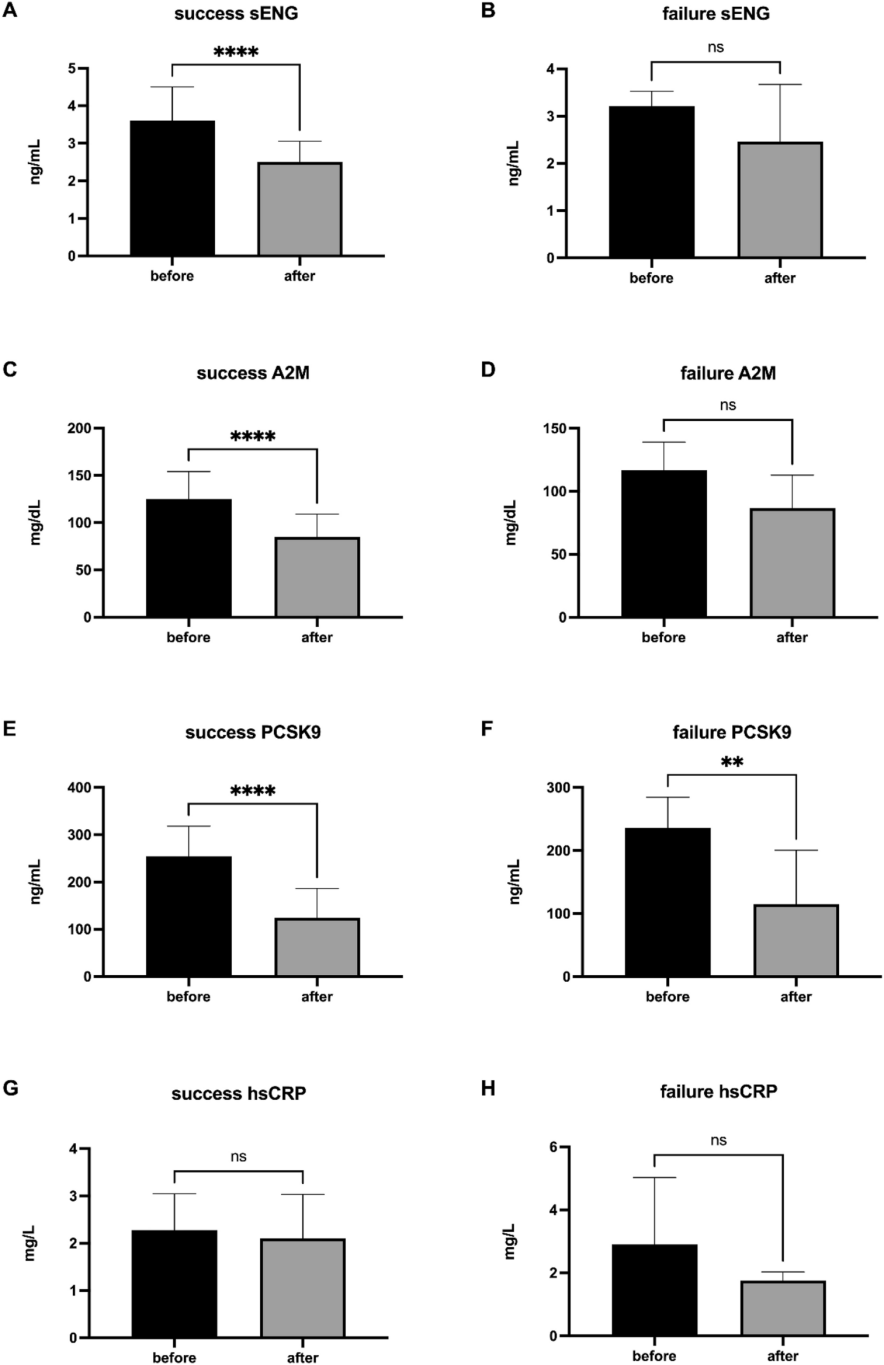
sENG, PCSK9, and markers of inflammation in patients with therapeutic success and therapeutic failure. Success ENG (**A**), failure ENG (**B**), success A2M (**C**), failure A2M (**D**), success PCSK9 (**E**) failure PCSK9 (**F**), success hsCRP (**G**), failure hsCRP (**H**) data are shown as median with interquartile range. Wilcoxon matched-paired signed rank tests were used in the first (1st), the second (2nd), and the third (3rd) rheopheresis treatment before and after therapy, ^ns^ *p* ≥ 0.05, **p < 0.01, ****p < 0.0001.

As assigned by ophthalmological assessment (Fig. 4), sENG, markers of inflammation, and PCSK9 before and after rheopheresis procedures were evaluated in the group of successfully treated patients, and the group of therapeutic failure. Rheopheresis procedures had a significant impact on lowering sENG (Fig. 4A), A2M (Fig. 4C), and PCSK9 (Fig. 4E) in successfully treated patients. In patients with therapeutic failure, the rheopheresis procedures had a generally milder effect on the analyzed markers, with a significant impact only in lowering PCSK9 (Fig. 4F) levels. In contrast, in the group of patients with therapeutic failure, the levels of sENG (Fig. 4B) and A2M (Fig. 4D) were not affected by the procedure at all. Rheopheresis procedures also did not affect the hsCRP levels in successfully treated patients (Fig. 4G) and patients with therapeutic failure (Fig. 4H).

## Discussion

This paper aimed to analyze data collected in the last 9 years. In patients with AMD treated with rheopheresis, we wanted to elucidate the benefit of this procedure with respect to plasma lipids, sENG levels, PCSK9, and selected biomarkers of inflammation.

We hypothesized that successful rheopheresis (with ophthalmologically confirmed AMD improvement) would be associated with positive changes in sENG, PSCK9, A2M, and hs-CRP levels. Here, for the first time, we demonstrate that sENG and A2M levels were reduced exclusively in successfully treated AMD patients after rheopheresis treatment.

AMD is characterized by clinically recognizable ocular findings in the choroid, retina, and retinal pigment epithelium that can lead to blindness. Although there are no universally accepted diagnostic criteria, AMD has most often been defined as the presence of drusen, RPE disturbance, geographic atrophy, RPE detachment, subretinal or choroidal neovascularization, and/or disciform scarring^35^.

Rheopheresis is considered an effective method for treating the dry form of AMD, as demonstrated by the number of studies^23,36^. Indeed, in this study, we showed that treatment with rheopheresis was associated with the long-lasting, significant, and desirable improvement of visual acuity, concomitantly with the decrease in the original area of pathological changes (DPED, area of soft drusen, area of RPE-atrophy), in 19 (73.1%) from the total of 26 patients who completed rheopheresis treatment using the predetermined scheme. In contrast, therapeutic failure was present in seven (26.9%) patients.

The beneficial effects of rheopheresis treatment were associated with a significant decrease in total cholesterol, LDL-C, HDL-C, apoprotein B, and lipoprotein (a) levels in AMD patients after all rheophereses during the monitored interval of 9 years.

Interestingly, several studies suggested a possible relation between AMD and risk factors of cardiometabolic disorders, including smoking, arterial hypertension, hypercholesterolemia, and type 2 diabetes mellitus ^35,37^.

It was demonstrated that sENG is a potential biomarker of hypercholesterolemia^11^, familial hypercholesterolemia^38^, endothelial dysfunction^39^, atherogenesis^40^ , and type 2 diabetes mellitus complications^8^. Indeed, sENG reduction was related to long-term successful lipoprotein apheresis in familial hypercholesterolemia patients^24^. In this study, we showed that despite a significant drop in lipid parameters in both successfully and unsuccessfully treated patients, sENG levels were significantly reduced exclusively in successfully treated AMD patients, possibly reflecting endothelial dysfunction improvement. This indicates that sENG reduction might reflect a decrease in cholesterol levels and even be a beneficial tool in AMD progression prevention.

In addition, inflammation could play an important role in AMD progression. hsCRP was shown to be a risk factor related to cardiovascular disorders, including atherosclerosis^41^. Moreover, hs-CRP levels were elevated in patients with a wet form of AMD^42^. Surprisingly, rheopheresis treatment did not significantly affect hs-CRP levels, and thus, hs-CRP was not related to successful AMD treatment in this study.

A2M is able to inhibit a broad spectrum of proteases, including ECM-remodeling matrix metalloproteinases^43^. It was demonstrated that A2M is expressed by RPE/choroidal endothelial cells^7^. Moreover, cleaved A2M could interact with macrophage-expressed LRP1 and modulate the inflammatory response^44^, suggesting its role in RPE/choroid inflammation^7^. It was demonstrated that A2M was reduced after rheopheresis, which was related to a drop in plasma and whole blood viscosity^25^. In this study, we showed that A2M was significantly reduced only in successfully treated patients with AMD, suggesting the potential effect of A2M reduction on rheological parameters of blood and inflammation.

There are several limitations of the study. Our study had a small sample size, including only the treatment group, and we did not incorporate a control treatment arm in this study. Based on the results of the ophthalmological examination, the patients were compared as the subjects who were clinically successfully treated and/or the individuals with therapeutic failure, which shortened the number of evaluated patients. The small number of participants in the present study may affect the accuracy of our results. Furthermore, since the rheopheresis treatment technique was recently carried out only in our medical centers in the Czech Republic because of technical and economic reasons, many AMD patients are unable to receive rheopheresis treatment, resulting in a particular bias in patient selection. Moreover, diagnosis of AMD disease in the early stage of the dry form is important but is not frequently possible. Our study’s strength is the collection of a large set of data comprising the long-term monitored interval of 9 years. We have experience with rheopheresis therapy in AMD patients in our center for more than 12 years. The total number of treated patients is 74, and in 66 patients the PCSK9 concentration was evaluated since 2012. To assess the outcome of AMD, it is necessary to respect its slow progression rate. Therefore, we set up 5-year follow-up intervals and finally evaluated 31 patients who completed it. Finally, our study group is unique also because of the evaluation of the relationship of PCSK9 concentration, markers of endothelial dysfunction, inflammation, and rheologically important parameters to the AMD prognosis. The valuable results obtained in this study will be evolved in our further research.

In conclusion, we demonstrated that most AMD patients benefit from the long-term effects of rheopheresis, specifically defined as improvement in best-corrected visual acuity and morphological ocular findings. Moreover, despite the substantial drop in lipid parameters in all treated patients, we showed for the first time that sENG and A2M could be potentially sensitive biomarkers of successful rheopheresis procedures. This also suggests that both biomarkers are relevant targets for AMD treatment.

## Data Availability

Data will be made available upon reasonable request to the corresponding author. The authors do not intend to share individual de-identified participant data.

## Abbreviations

PCSK9: Proprotein convertase subtilisin kexin 9
sENG: Soluble endoglin
A2M: Alpha-2-macroglobulin
hsCRP: High-sensitivity C-reactive protein
AMD: Age-related macular degeneration
DPED: Drusenoid pigment epithelium detachment
BCVA: Best-corrected visual acuity
ERG: Electroretinography
TC: Total cholesterol
LDL: Low-density lipoprotein
LDL-C: Low-density lipoprotein cholesterol
HDL-C: High-density lipoprotein cholesterol
apoB: Apoprotein B
Lp (a): Lipoprotein (a)

## References

[1] Jager, R. D., Mieler, W. F. & Miller, J. W. Age-related macular degeneration. *N. Engl. J. Med.* **358**, 2606–2617 (2008). 18550876 10.1056/NEJMra0801537

[2] Roizenblatt, M., Naranjit, N., Maia, M. & Gehlbach, P. L. The question of a role for statins in age-related macular degeneration. *Int. J. Mol. Sci.* **19**, 3688 (2018). 30469381 10.3390/ijms19113688 PMC6274767

[3] Tserentsoodol, N. et al. Uptake of cholesterol by the retina occurs primarily via a low density lipoprotein receptor-mediated process. *Mol. Vis.* **12**, 1306–1318 (2006). 17110914

[4] Fliesler, S. J., Florman, R., Rapp, L. M., Pittler, S. J. & Keller, R. K. In vivo biosynthesis of cholesterol in the rat retina. *FEBS Lett.* **335**, 234–238 (1993). 8253203 10.1016/0014-5793(93)80736-e

[5] Tserentsoodol, N. et al. Intraretinal lipid transport is dependent on high density lipoprotein-like particles and class B scavenger receptors. *Mol. Vis.* **12**, 1319–1333 (2006). 17110915

[6] Klein, R. et al. Markers of inflammation, oxidative stress, and endothelial dysfunction and the 20-year cumulative incidence of early age-related macular degeneration: the Beaver Dam Eye Study. *JAMA Ophthalmol.* **132**, 446–455 (2014). 24481424 10.1001/jamaophthalmol.2013.7671 PMC4076038

[7] Lehmann, G. L. et al. Retinal pigment epithelium-secreted VEGF-A induces alpha-2-macroglobulin expression in endothelial cells. *Cells* **11**, 2975 (2022). 36230937 10.3390/cells11192975 PMC9564307

[8] Blazquez-Medela, A. M. et al. Increased plasma soluble endoglin levels as an indicator of cardiovascular alterations in hypertensive and diabetic patients. *BMC Med.* **8**, 86 (2010). 21171985 10.1186/1741-7015-8-86 PMC3012013

[9] Kaitu’u-Lino, T. J. et al. MMP-14 is expressed in preeclamptic placentas and mediates release of soluble endoglin. *Am. J. Pathol.* **180**, 888–894 (2012). 22296769 10.1016/j.ajpath.2011.11.014

[10] Blaha, M. et al. Elevated serum soluble endoglin (sCD105) decreased during extracorporeal elimination therapy for familiar hypercholesterolemia. *Atherosclerosis* **197**, 264–270 (2008). 17540382 10.1016/j.atherosclerosis.2007.04.022

[11] Blann, A. D., Wang, J. M., Wilson, P. B. & Kumar, S. Serum levels of the TGF-beta receptor are increased in atherosclerosis. *Atherosclerosis* **120**, 221–226 (1996). 8645363 10.1016/0021-9150(95)05713-7

[12] Malhotra, R. et al. Circulating angiogenic modulatory factors predict survival and functional class in pulmonary arterial hypertension. *Pulm. Circ.* **3**, 369–380 (2013). 24015338 10.4103/2045-8932.110445 PMC3757832

[13] Leanos-Miranda, A. et al. Soluble endoglin as a marker for preeclampsia, its severity, and the occurrence of adverse outcomes. *Hypertension* **74**, 991–997 (2019). 31446801 10.1161/HYPERTENSIONAHA.119.13348

[14] Rathouska, J., Jezkova, K., Nemeckova, I. & Nachtigal, P. Soluble endoglin, hypercholesterolemia and endothelial dysfunction. *Atherosclerosis* **243**, 383–388 (2015). 26520890 10.1016/j.atherosclerosis.2015.10.003

[15] Vitverova, B. et al. Soluble endoglin and hypercholesterolemia aggravate endothelial and vessel wall dysfunction in mouse aorta. *Atherosclerosis* **271**, 15–25 (2018). 29459262 10.1016/j.atherosclerosis.2018.02.008

[16] Klingel, R., Fassbender, C., Fassbender, T. & Gohlen, B. Clinical studies to implement Rheopheresis for age-related macular degeneration guided by evidence-based-medicine. *Transfus. Apher. Sci.* **29**, 71–84 (2003). 12877897 10.1016/S1473-0502(03)00101-0

[17] Borberg, H. & Tauchert, M. Rheohaemapheresis of ophthalmological diseases and diseases of the microcirculation. *Transfus. Apher. Sci.* **34**, 41–49 (2006). 16343990 10.1016/j.transci.2005.09.001

[18] Zion, I. B. et al. Pulsatile ocular blood flow: relationship with flow velocities in vessels supplying the retina and choroid. *Br. J. Ophthalmol.* **91**, 882–884 (2007). 17576711 10.1136/bjo.2006.108340 PMC1955661

[19] Klingel, R. et al. RheoNet registry analysis of rheopheresis for microcirculatory disorders with a focus on age-related macular degeneration. *Ther. Apher. Dial.* **14**, 276–286 (2010). 20609179 10.1111/j.1744-9987.2010.00807.x

[20] Langrova, H. et al. Rheopheresis in the treatment of age-related macular degeneration. *Cesk. Slov. Oftalmol.* **79**, 8–24 (2022). 36858957 10.31348/2023/2

[21] Rencova, E. et al. Reduction in the drusenoid retinal pigment epithelium detachment area in the dry form of age-related macular degeneration 2.5 years after rheohemapheresis. *Acta Ophthalmol.* **91**, e406-408 (2013). 22971248 10.1111/j.1755-3768.2012.02503.x

[22] Schwartz, J. et al. Guidelines on the use of therapeutic apheresis in clinical practice-evidence-based approach from the writing committee of the American society for apheresis: the sixth special issue. *J. Clin. Apher.* **28**, 145–284 (2013). 23868759 10.1002/jca.21276

[23] Studnicka, J. et al. Long-term outcomes of rheohaemapheresis in the treatment of dry form of age-related macular degeneration. *J. Ophthalmol,* **2013**, 135798 (2013). 24455194 10.1155/2013/135798 PMC3880698

[24] Visek, J. et al. Monitoring of up to 15 years effects of lipoprotein apheresis on lipids, biomarkers of inflammation, and soluble endoglin in familial hypercholesterolemia patients. *Orphanet. J. Rare Dis.* **16**, 110 (2021). 33640001 10.1186/s13023-021-01749-w PMC7913462

[25] Blaha, M. et al. Changes of the complement system and rheological indicators after therapy with rheohemapheresis. *Atheroscler. Suppl.* **18**, 140–145 (2015). 25936318 10.1016/j.atherosclerosissup.2015.02.009

[26] Tavori, H., Giunzioni, I., Linton, M. F. & Fazio, S. Loss of plasma proprotein convertase subtilisin/kexin 9 (PCSK9) after lipoprotein apheresis. *Circ. Res.* **113**, 1290–1295 (2013). 24122718 10.1161/CIRCRESAHA.113.302655 PMC3939022

[27] Augood, C. et al. Methods for a population-based study of the prevalence of and risk factors for age-related maculopathy and macular degeneration in elderly European populations: the EUREYE study. *Ophthalmic Epidemiol.* **11**, 117–129 (2004). 15255027 10.1076/opep.11.2.117.28160

[28] Rencova, E. et al. Haemorheopheresis could block the progression of the dry form of age-related macular degeneration with soft drusen to the neovascular form. *Acta Ophthalmol.* **89**, 463–471 (2011). 20102350 10.1111/j.1755-3768.2009.01710.x

[29] Blaha, M. et al. Rheohaemapheresis in the treatment of nonvascular age-related macular degeneration. *Atheroscler. Suppl.* **14**, 179–184 (2013). 23357162 10.1016/j.atherosclerosissup.2012.10.023

[30] Schwartz, J. et al. Guidelines on the use of therapeutic apheresis in clinical practice-evidence-based approach from the writing committee of the American society for apheresis: The seventh special issue. *J. Clin. Apher.* **31**, 149–162 (2016). 27322218 10.1002/jca.21470

[31] Blaha, V. et al. Antioxidant defense system in familial hypercholesterolemia and the effects of lipoprotein apheresis. *Atheroscler. Suppl.* **30**, 159–165 (2017). 29096832 10.1016/j.atherosclerosissup.2017.05.002

[32] Pulido, J. S., Multicenter Investigation of Rheopheresis for AMDSG. Multicenter prospective, randomized, double-masked, placebo-controlled study of Rheopheresis to treat nonexudative age-related macular degeneration: interim analysis. *Trans. Am. Ophthalmol. Soc.* **100**, 85–106 (2002). 12545682 PMC1358951

[33] Blaha, M. et al. Extracorporeal LDL cholesterol elimination (25 years of experience in CZ). *Atheroscler. Suppl.* **10**, 17–20 (2009). 20129368 10.1016/S1567-5688(09)71804-5

[34] Rencova, E. et al. Preservation of the photoreceptor inner/outer segment junction in dry age-related macular degeneration treated by rheohemapheresis. *J. Ophthalmol.* **2015**, 359747 (2015). 26351571 10.1155/2015/359747 PMC4553324

[35] Snow, K. K. & Seddon, J. M. Do age-related macular degeneration and cardiovascular disease share common antecedents?. *Ophthalmic Epidemiol.* **6**, 125–143 (1999). 10420212 10.1076/opep.6.2.125.1558

[36] Connelly-Smith, L. et al. Guidelines on the use of therapeutic apheresis in clinical practice—evidence-based approach from the writing committee of the American society for apheresis: The ninth special issue. *J. Clin. Apher.* **38**, 77–278 (2023). 37017433 10.1002/jca.22043

[37] Pennington, K. L. & DeAngelis, M. M. Epidemiology of age-related macular degeneration (AMD): associations with cardiovascular disease phenotypes and lipid factors. *Eye Vis.* **3**, 34 (2016). 10.1186/s40662-016-0063-5 PMC5178091 28032115

[38] Blaha, M. et al. Elevated serum soluble endoglin (sCD105) decreased during extracorporeal elimination therapy for familial hypercholesterolemia. *Atherosclerosis* **197**, 264–270 (2008). 17540382 10.1016/j.atherosclerosis.2007.04.022

[39] Vicen, M. et al. Regulation and role of endoglin in cholesterol-induced endothelial and vascular dysfunction in vivo and in vitro. *FASEB J.* **33**, 6099–6114 (2019). 30753095 10.1096/fj.201802245R

[40] Strasky, Z. et al. Cholesterol effects on endoglin and its downstream pathways in ApoE/LDLR double knockout mice. *Circ. J.* **75**, 1747–1755 (2011). 21576826 10.1253/circj.cj-10-1285

[41] Calabro, P., Golia, E. & Yeh, E. T. CRP and the risk of atherosclerotic events. *Semin. Immunopathol.* **31**, 79–94 (2009). 19415283 10.1007/s00281-009-0149-4

[42] Shijo, T. et al. Association of CRP levels with ARMS2 and CFH variants in age-related macular degeneration. *Int. Ophthalmol.* **40**, 2735–2742 (2020). 32507953 10.1007/s10792-020-01460-y

[43] Vandooren, J. & Itoh, Y. Alpha-2-macroglobulin in inflammation immunity and infections. *Front. Immunol.* **12**, 803244 (2021). 34970276 10.3389/fimmu.2021.803244 PMC8712716

[44] Mantuano, E. et al. LDL receptor-related protein-1 regulates NFkappaB and microRNA-155 in macrophages to control the inflammatory response. *Proc. Natl. Acad. Sci. U. S. A.* **113**, 1369–1374 (2016). 26787872 10.1073/pnas.1515480113 PMC4747752

